# Antimicrobial Resistance in *Acinetobacter baumannii* Isolated from Ready-to-Eat Foods in Saudi Arabia

**DOI:** 10.3390/pathogens15030261

**Published:** 2026-03-01

**Authors:** Eman Marzouk, Adil Abalkhail

**Affiliations:** Department of Public Health, College of Applied Medical Sciences, Qassim University, P.O. Box 6666, Buraydah 51452, Saudi Arabia; e.marzouk@qu.edu.sa

**Keywords:** antibiotic resistance, carbapenemases, multidrug resistance, MALDI-TOF MS, real-time PCR, VITEK-2, foodborne bacteria, One Health

## Abstract

*Acinetobacter baumannii* is widely recognized as a problematic pathogen in healthcare settings due to its ability to acquire resistance to multiple antimicrobial agents. However, less attention has been given to its presence outside hospitals. In this cross-sectional, laboratory-based surveillance study, we investigated the occurrence of *A. baumannii* in ready-to-eat (RTE) foods sold at retail outlets in four cities of the Al-Qassim region, Saudi Arabia, during a single season. A total of 240 RTE food samples were analyzed using culture-based and molecular approaches for species confirmation, and antimicrobial susceptibility profiles were determined. *A. baumannii* was identified in 19 samples (7.9%), spanning several food categories. Most isolates showed resistance to multiple antimicrobial classes, and 16 met the criteria for multidrug resistance (MDR). Among the confirmed isolates, *bla*_OXA-23-like_ was detected in 16 (84.2%), *bla*_OXA-24/40-like_ in 2 (10.5%), and *bla*_OXA-58-like_ in 1 (5.3%). Resistance to fluoroquinolones, tetracyclines, and aminoglycosides was common, and OXA-type carbapenemase genes were detected in 16 isolates. These findings indicate that RTE foods can represent non-clinical environments in which MDR *A. baumannii* may be detected. Including food sources in antimicrobial resistance surveillance may therefore strengthen our understanding of the ecology of this pathogen within a One Health framework.

## 1. Introduction

Data on *Acinetobacter baumannii* in ready-to-eat (RTE) foods remain limited in the Middle East. Non-clinical environments are increasingly recognized as part of the broader ecology of antimicrobial resistance (AMR). However, region-specific evidence on the occurrence and resistance profiles of *A. baumannii* in RTE foods is scarce when compared with the extensive hospital-based literature.

This gap limits our understanding of the potential role of foods as non-clinical reservoirs of resistant bacteria. It also constrains the development of integrated One Health surveillance strategies. Addressing this gap requires systematic data on foodborne occurrence, resistance patterns, and the performance of commonly used identification methods in food matrices.

*Acinetobacter baumannii* is a multidrug-resistant (MDR) Gram-negative bacterium that has attracted global attention because of its association with severe healthcare-associated infections [1,2]. Carbapenem-resistant *A. baumannii* (CRAB) is classified by the World Health Organization as a “Critical” priority pathogen, reflecting its high resistance potential and limited treatment options [3]. Beyond clinical settings, this organism can persist in diverse environments. This persistence is supported by tolerance to desiccation and the ability to form biofilms on abiotic surfaces [4,5].

Within the One Health framework, AMR is understood to circulate across human, animal, food, and environmental compartments. This interdependence requires coordinated surveillance across sectors [6,7,8]. Accordingly, increasing attention has shifted toward the detection of *A. baumannii* outside hospitals. Such detection has been reported in water, soil, and food matrices. In these environments, the organism may persist without causing immediate disease while still harboring clinically relevant resistance traits [9].

Studies from different regions have reported *Acinetobacter* species, including *A. baumannii*, in raw and RTE foods. These foods include meat, seafood, and fresh produce, and isolates often exhibit MDR [10,11,12]. Ababneh et al. [12] identified *A. baumannii* in fresh produce with strong biofilm-forming capacity and extensively drug-resistant phenotypes. These findings support concerns regarding persistence along the food chain. Similarly, Carvalheira et al. [9] documented the widespread presence of *Acinetobacter* spp. in foods and drinking water. Surveys using matrix-assisted laser desorption/ionization time-of-flight mass spectrometry (MALDI-TOF MS) have also reported the frequent detection of the *A. calcoaceticus baumannii* (ACB) complex in retail food samples [13]. In addition, food-associated *Acinetobacter* strains can tolerate commonly used sanitizers. These include sodium hypochlorite and peracetic acid, which complicates their control in food-processing environments [14].

Despite these reports, most available studies have focused primarily on detection and resistance prevalence. Few have evaluated diagnostic performance, method agreement, or operational metrics such as testing time and cost. This limitation is particularly evident in the Middle East and North Africa, where systematic surveillance of RTE foods for *A. baumannii* remains sparse compared with hospital-based datasets [9]. International food-safety and public-health bodies have emphasized the need for integrated AMR monitoring within the food sector. They have also highlighted the importance of region-specific data to inform surveillance design and risk assessment [15,16].

Reliable identification of *A. baumannii* is central to such surveillance efforts, particularly in food matrices where closely related *Acinetobacter* species may coexist. Rapid proteomic approaches, such as MALDI-TOF MS, are widely used in clinical microbiology. These platforms offer clear advantages for high-throughput and large-scale surveillance [17,18]. However, their comparative performance and practical utility in food-sector surveillance have been less thoroughly examined, especially when benchmarked against molecular confirmation methods [19]. In clinical microbiology, MALDI-TOF MS provides reliable identification when supported by updated databases and performs comparably to molecular confirmation assays targeting genes such as *bla*_OXA-51-like_, *gyrB*, and *rpoB*. However, the performance of these tools in food matrices has been less extensively evaluated [19].

CRAB is mediated by multiple mechanisms. The most important mechanism is the production of carbapenem-hydrolyzing β-lactamases, particularly class D OXA-type enzymes, which are widely distributed globally [20,21]. Class B metallo-β-lactamases, including NDM, VIM, and IMP, have also been reported, although they occur less frequently than OXA-type enzymes, while class A carbapenemases are rare in this species [20,22]. In addition to carbapenemase production, non-enzymatic mechanisms contribute to resistance, including reduced outer-membrane permeability due to porin loss or the modification and overexpression of multidrug efflux pumps [23,24]. These mechanisms may act alone or in combination, resulting in high-level carbapenem resistance in clinical, environmental, and food-associated isolates [20,21].

Globally, CRAB is predominantly mediated by OXA-type β-lactamases. Among these, *bla*_OXA-23_ is most frequently reported, with sporadic detection of metallo-β-lactamases such as *bla*_NDM_ [25,26,27,28]. In antimicrobial susceptibility testing (AST), reference standards such as CLSI M52 and M100 provide guidance for verification procedures. These documents define acceptable performance thresholds for nonfermenting Gram-negative bacteria [29]. Under validated conditions, published studies of Gram-negative workflows report categorical agreement (CA) near or above 90%. These findings support the feasibility of structured method-comparison approaches to guide local platform selection [30,31].

In this context, the present study applied a One Health approach to investigate the occurrence of *A. baumannii* in RTE foods. The primary focus was to estimate prevalence and characterize AMR patterns in retail RTE foods from four cities in the Al-Qassim region, Saudi Arabia.

Standard laboratory workflows were used to support accurate detection and confirmation. Comparative evaluation of identification and susceptibility testing approaches was performed to ensure analytical robustness, but these analyses were secondary to the surveillance objectives.

The study was designed to address three objectives: (i) to determine the prevalence of MDR *A. baumannii* in retail RTE foods; (ii) to confirm species identity using molecular methods; and (iii) to describe antimicrobial susceptibility profiles using defined performance criteria [19,29,32,33].

## 2. Materials and Methods

A cross-sectional, laboratory-based surveillance study of RTE foods was carried out in four cities of the Al-Qassim region: Buraydah, Unaizah, Al-Bukayriyah, and Ar Rass. These sites were selected because of their high density of food-service establishments. This approach was intended to reflect everyday consumer exposure. Throughout the sampling period, items available in local markets were purchased anonymously. Samples were transported to the laboratory under cold-chain conditions. This process provided a realistic snapshot of potential microbial contamination along the food consumer interface within a One Health framework. The study design and reporting followed the Standards for Reporting Diagnostic Accuracy (STARD) guidance. Reference standards, index tests, and analysis plans were defined a priori.

To characterize MDR *A. baumannii*, a stepwise, multi-platform laboratory workflow was implemented using standard microbiological procedures described elsewhere. Isolates were initially screened using routine culture and biochemical methods. These were then identified using the VITEK-2 Compact automated system. MALDI-TOF MS (MALDI Biotyper, MBT) was subsequently applied to improve discrimination within the ACB complex. Species confirmation was performed by real-time PCR on the 7500 Fast platform using established SYBR Green assays targeting *bla*_OXA-51-like_. Additional targets, including *gyrB* or *rpoB*, were amplified only when further resolution was required.

**Figure 1:**
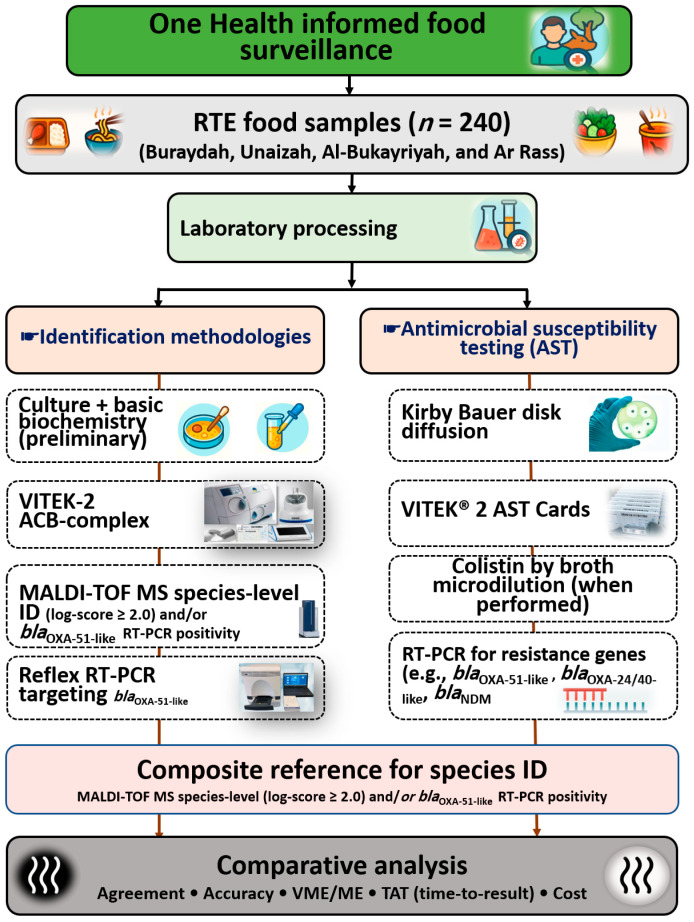
Workflow for laboratory detection and AST of *A. baumannii* in RTE foods. Isolates were recovered by culture and biochemistry, screened by VITEK-2 Compact, identified by MALDI-TOF MS and real-time polymerase chain reaction (RT-PCR), and tested for antimicrobial susceptibility using Kirby Bauer disk diffusion and VITEK-2, with colistin by broth microdilution. Composite species identity required either MALDI-TOF MS species-level scoring or *bla*_OXA-51-like_ RT-PCR positivity.

Confirmed isolates underwent AST using standard Kirby Bauer disk diffusion and VITEK-2 automated AST Gram-negative cards. These methods were applied to characterize AST profiles and operational feasibility, including turnaround time. AST interpretation followed current CLSI M100 (35th edition) breakpoints, with method verification guided by CLSI M52 [29,34]. Routine quality control procedures and operational steps followed the CLSI and manufacturer recommendations and are therefore not reiterated here. Figure 1 presents an overview of the analytical workflow, including sampling, identification, AST, and the integrated application of phenotypic, proteomic, and molecular methods.

### 2.1. Study Setting, Sampling Frame, and Sample Size

Between March and June 2025, 240 RTE food samples were collected from four cities in the Al-Qassim region: Buraydah, Unaizah, Al-Bukayriyah, and Ar Rass. A stratified sampling framework was applied by city and food category to ensure coverage of commonly consumed RTE items. Within each food category, sample selection at retail outlets was convenience-based. This approach reflected products available at the time of purchase and typical consumer exposure. From each city, 60 samples were collected across six predefined food categories: salads, meat-based dishes, poultry items, dairy-based dishes, rice dishes, and desserts. This resulted in equal numbers per category.

Samples were further distributed across food service types: street vendors (*n* = 100), quick-service restaurants (*n* = 70), and bakeries (*n* = 70). All items were purchased anonymously during routine service hours. Samples were placed in sterile containers and transported to the laboratory under cold chain conditions following standard food microbiology handling practices. Delivery to the laboratory occurred within 6–8 h. Metadata recorded at purchase included food category, outlet type, holding temperature, packaging form, and time of purchase.

The use of equal numbers per food category was intentional. It was designed to facilitate balanced comparisons across food categories and diagnostic approaches, rather than reflect true market share or consumption frequency. Accordingly, prevalence estimates represent category-specific observations within the study frame and should not be interpreted as population-level exposure estimates. In addition, sampling was limited to four cities within a single region and one season. Therefore, the findings are not nationally representative of RTE foods in Saudi Arabia.

In the laboratory, each item (≥100 g or mL) was processed using standard procedures for RTE food microbiology as described in international guidelines. A 25 g test portion was homogenized in 225 mL of sterile diluent (1:9) using stomacher bags, in accordance with ISO 6887-1 and the U.S. Food and Drug Administration (FDA) Bacteriological Analytical Manual (BAM), Chapter 1, recommendations for RTE foods [35]. The sample size was selected to estimate occurrence with an expected precision of approximately ±4–5 percentage points at the 95% confidence level. It also allowed for estimation of diagnostic sensitivity and specificity with confidence interval widths of approximately ±10 percentage points.

Only foods intended for direct consumption without further cooking were included. Samples were required to meet safe temperature criteria (cold ≤ 5 °C or hot ≥ 57 °C). All items were within the labeled shelf life when applicable and were obtained as unopened retail packages or freshly served portions. Each sample met the minimum weight requirement to allow replicate testing. These inclusion and exclusion criteria followed the FDA Food Code 2022 and related guidance and are therefore not described in further procedural detail [36]. Samples were excluded if they were not truly RTE, insufficient in size, exposed to temperature deviations during transport, or associated with damaged packaging or incomplete metadata. All accepted samples were maintained under controlled cold chain conditions until analysis, following established guidance to ensure analytical reliability [37].

### 2.2. Isolation and Presumptive Identification

From the 10^−1^ homogenate and serial dilutions, 100 μL aliquots were plated on CHROMagar *Acinetobacter* (with or without MDR supplement; CHROMagar, Paris, France) and MacConkey agar (Oxoid, Basingstoke, UK). Plates were incubated at 35–37 °C for 24–48 h following standard culture conditions for non-fermenting Gram-negative bacteria.

Presumptive isolates were characterized using routine Gram staining and biochemical screening consistent with established identification criteria for *Acinetobacter* spp., rather than through exhaustive phenotypic testing. The choice of CHROMagar medium was based on its validated performance for selective isolation and presumptive differentiation of *A. baumannii* and related species [38]. This initial culture-based screening step ensured that downstream identification and resistance analyses were performed on pure, viable isolates.

### 2.3. Automated Phenotypic Identification (VITEK-2 Compact)

Presumptive colonies were prepared for automated identification using standard procedures recommended by the manufacturer, including subculture and suspension in sterile saline adjusted to the required turbidity. Gram-negative identification (GN ID) cards (bioMérieux, Marcy-l’Étoile, France) were used to generate biochemical profiles through automated kinetic readings, as described in established clinical microbiology protocols [39,40,41].

The VITEK-2 Compact system provided identification at the ACB complex level, together with confidence categories (excellent, good, or low discrimination) and possible alternative assignments. Isolates yielding excellent or good confidence results were accepted as definitive ACB-complex identifications for descriptive analysis. Results reported as low discrimination were classified as non-definitive and were not excluded. All low-discrimination results and any discordant identifications were resolved using MALDI-TOF MS and/or species-specific RT-PCR according to a predefined identification algorithm.

For method-comparison analyses, low-discrimination VITEK-2 results were treated conservatively and counted as incorrect when they did not agree with the composite reference standard. Routine quality control procedures are described in Section 2.10. Operators were blinded to the RT-PCR findings during VITEK-2 testing. Typical identification turnaround times ranged from 6 to 8 h.

### 2.4. MALDI-TOF MS Identification (MBT)

#### 2.4.1. Sample Preparation and Acquisition

Fresh colonies were processed for MALDI-TOF MS identification using standard manufacturer-recommended procedures and previously validated protocols [42,43,44]. Briefly, 2–3 colonies were applied to a polished steel target plate, air-dried, and overlaid with an α-cyano-4-hydroxycinnamic acid (HCCA) matrix (Bruker Daltonics, Bremen, Germany). Ethanol–formic acid extraction was performed only when spectra quality was insufficient, as indicated by low log-scores.

Spectra were acquired using a Bruker Microflex LT (MALDI-TOF MS) system operated in linear positive mode. Instrument calibration and verification procedures are described in Section 2.10. Data acquisition was performed using FlexControl within the Compass Flex Series version 1.3. Spectra were matched against the Bruker MALDI Biotyper reference database [42,43,44]. Analysts were blinded to RT-PCR outcomes during spectral interpretation.

#### 2.4.2. Scoring and Data Analysis

Log-score interpretation followed established manufacturer thresholds. Scores ≥ 2.0 were considered indicative of species-level identification. Scores between 1.70 and 1.99 were interpreted at the genus level, while scores < 1.70 were considered unreliable. Unreliable or ambiguous results were reanalyzed or confirmed by RT-PCR, in accordance with the predefined identification workflow.

Multivariate analyses, including principal component analysis (PCA) and main spectra profile (MSP) generation, were applied following established MALDI-TOF MS validation approaches to assess spectral consistency and clustering. Outlier spectra were re-extracted or retested as needed.

### 2.5. Molecular Species Confirmation by Real-Time PCR

DNA was extracted using standard silica-column or boiling lysis methods described previously. Real-time PCR was performed on the 7500 Fast platform using validated SYBR Green protocols [45,46,47]. All reactions were run in duplicate, and only concordant results were accepted. Melt-curve analysis was used to confirm amplification specificity. Each run included positive controls (*A. baumannii* ATCC 19606), non-target controls, and no-template controls.

Species confirmation targeted the intrinsic *bla*_OXA-51-like_ gene. Because reliance on a single marker may not capture rare or atypical members of the *ACB* complex, additional targets (*gyrB* or *rpoB*) were amplified only when needed to resolve ambiguous or discordant results. This approach was used to support conservative species assignment rather than exhaustive molecular characterization. Isolates identified as bacteria other than *A. baumannii*, including non-*baumannii Acinetobacter* species, were excluded from further analysis, consistent with the study’s focus.

### 2.6. Detection of Carbapenem Resistance Genes by Real-Time PCR

Confirmed *A. baumannii* isolates were screened for carbapenem resistance genes using real-time PCR. The panel included OXA-type (*bla*_OXA-23-like_, *bla*_OXA-24/40-like_, *bla*_OXA-58-like_) and metallo-β-lactamases (*bla*_NDM_, *bla*_VIM_, *bla*_IMP_), using previously published primer sets and amplification conditions. Previously published primers and amplification conditions were used (Table 1).

The resistance gene panel was intentionally targeted to carbapenemases most commonly reported in *A. baumannii* in food, environmental, and regional clinical settings; rare OXA variants (e.g., *bla*_OXA-143-like_, *bla*_OXA-258-like_) and class A carbapenemases (e.g., *bla*_KPC_, *bla*_GES_) were not included. Primer sequences and annealing temperatures are summarized in Table 1.

### 2.7. Composite Reference Standard

For accuracy evaluation, an isolate was defined as *A. baumannii* using a composite reference approach based on established identification criteria. An isolate was considered confirmed if MALDI-TOF MS yielded a score ≥ 2.0 consistent with species-level identification and/or if RT-PCR targeting *bla*_OXA-51-like_ was positive. Discordant or ambiguous results were resolved using repeat testing or rpoB sequencing, following previously published approaches [55,56].

### 2.8. Antimicrobial Susceptibility Testing (AST)

AST was performed using both Kirby Bauer disk diffusion and the VITEK-2 Compact system, following CLSI-recommended procedures. For disk diffusion, isolates were standardized to the required turbidity and inoculated onto Mueller Hinton agar, incubated under routine conditions, and tested against a panel of antimicrobial agents including carbapenems, aminoglycosides, fluoroquinolones, tetracyclines, and trimethoprim–sulfamethoxazole. Colistin was not tested by disk diffusion, in accordance with CLSI guidance, and broth microdilution was applied selectively to resolve discrepant results [34]. Broth microdilution was applied selectively to resolve discrepant results. Ampicillin-sulbactam and cefiderocol were not included because these agents were not routinely available for food-sector susceptibility testing during the study period.

Confirmed isolates were additionally tested using the VITEK-2 Compact system with Gram-negative AST cards, following the manufacturer’s instructions and established laboratory protocols. Minimum inhibitory concentrations (MICs) and categorical interpretations were assigned according to the CLSI M100 criteria. Quality control procedures for AST are described in Section 2.10.

### 2.9. Multidrug-Resistance Definition

MDR was defined as non-susceptibility to at least one agent in three or more antimicrobial categories, and extensively drug-resistant (XDR) as non-susceptibility to at least one agent in all but one or two categories, in accordance with the international definitions proposed by Magiorakos et al. [57].

### 2.10. Quality Assurance

Quality assurance procedures followed CLSI guidance and manufacturer recommendations across all analytical platforms. MALDI-TOF MS runs included routine calibration using the Bruker Bacterial Test Standard (BTS). RT-PCR assays incorporated appropriate positive, non-target, and no-template controls. AST included reference strains (ATCC 25922 and ATCC 27853) for verification. Low-confidence or discordant identifications were resolved using MALDI-TOF MS and RT-PCR according to predefined criteria [43].

### 2.11. Data Management and Statistical Analysis

Prevalence estimates were calculated with corresponding 95% confidence intervals. Analyses focused on descriptive statistics and diagnostic outcomes. Statistical analyses were performed using R software (version 4) with the binom and epiR packages [29]. Comparative statistical testing between cities, food categories, or outlet types was intentionally not performed due to the study design and subgroup sizes.

### 2.12. Ethics and Biosafety

The study involved only food samples and did not include human subjects. Institutional authorization was obtained in accordance with local policy. All laboratory work involving *A. baumannii* was conducted under biosafety level 2 (BSL-2) conditions, and waste was handled and decontaminated following standard biosafety procedures.

## 3. Results

### 3.1. Sample Workflow

A total of 240 RTE food samples were screened using a stepwise workflow that combined selective culture, presumptive phenotypic assessment, proteomic identification, and molecular confirmation. Growth on selective media was observed in 49 samples, of which 43 yielded presumptive *Acinetobacter* spp. isolates based on colony morphology and basic biochemical characteristics. Subsequent automated identification assigned 27 isolates to the ACB complex.

Confirmatory testing further refined species attribution. Twenty-one isolates achieved a MALDI-TOF MS species-level score (≥2.0), while 19 isolates were confirmed as *A. baumannii* by RT-PCR targeting the intrinsic *bla*_OXA-51-like_ gene. These 19 isolates met the predefined composite reference standard and were included in downstream prevalence estimation and AMR analyses.

Presumptive *Acinetobacter* isolates that did not meet the composite confirmation criteria were excluded from downstream analyses, and their distribution across the screening workflow is summarized in Table S4. The numerical progression of samples and isolates across the workflow is summarized in Table 2.

### 3.2. Prevalence of A. baumannii in RTE Foods

Overall, 19 of 240 RTE food samples (7.9%; 95% CI: 4.9–12.1%) were detected in a subset of RTE food samples, indicating measurable contamination within the sampled retail food environment. Prevalence estimates showed modest variation across the four cities, with no pronounced geographic clustering observed within the study region.

When stratified by food category, *A. baumannii* was more frequently detected in salad samples (6/40), while lower detection frequencies were observed in other RTE food categories. These comparisons are descriptive only, as no formal statistical testing was performed to assess differences between categories.

**Figure 2:**
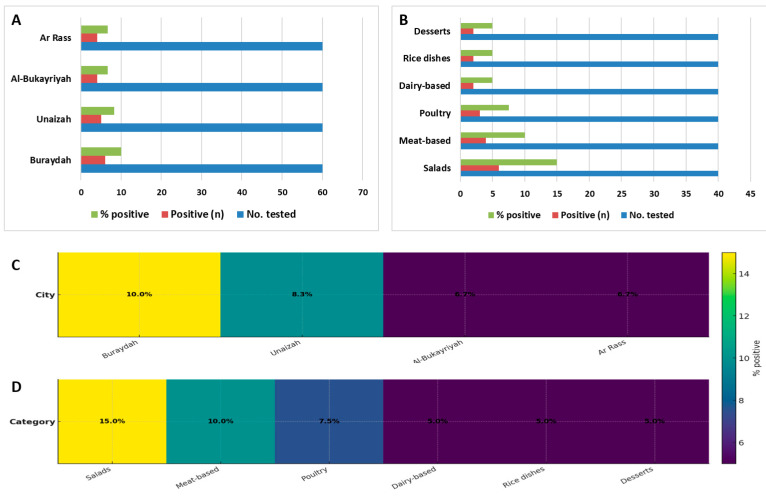
Prevalence patterns of composite-confirmed *A. baumannii* in RTE food samples by city and food category. (**A**,**B**) Bar charts summarize the number of samples tested, the count of positive samples, and the percentage positive for each city ((**A**): Buraydah, Unaizah, Al-Bukayriyah, Ar Rass) and food category ((**B**): salads, meat-based, poultry, dairy-based, rice dishes, desserts). (**C**,**D**) Heatmaps visualize the same prevalence data as a color gradient, where darker shades indicate higher positivity. City-level prevalence values were: Buraydah 10.0%, Unaizah 8.3%, Al-Bukayriyah 6.7%, and Ar Rass 6.7%. Category-level prevalence rates were: salads 15.0%, meat-based 10.0%, poultry 7.5%, dairy-based 5.0%, rice dishes 5.0%, and desserts 5.0%. These panels reveal heterogeneous contamination patterns and can inform targeted, risk-based food safety surveillance.

Detailed prevalence estimates by city and food category, including absolute numbers, percentages, and 95% confidence intervals, are provided in Table 3 and visualized in Figure 2.

### 3.3. Identification Performance

Identification accuracy was evaluated against a composite reference standard combining MALDI-TOF MS and RT-PCR. MALDI-TOF MS and RT-PCR were used to establish species confirmation, while automated phenotypic identification served as an initial screening step. Detailed analytical performance metrics and proteomic analyses are provided in the Supplementary Materials (Tables S1 and S2 and Figure S1).

Section 3 focuses on confirmed species attribution rather than comparative performance testing. Among the composite-confirmed isolates, MALDI-TOF MS consistently achieved species-level identification, and RT-PCR targeting *bla*_OXA-51-like_ showed full concordance with composite confirmation. These findings support the robustness of the integrated identification workflow for RTE food matrices.

Analysis of MALDI-TOF MS log-score distributions showed that most isolates achieved species-level confidence, with a smaller proportion yielding genus-level scores that required molecular confirmation. No isolates produced unreliable spectra. Agreement between MALDI-TOF MS confidence categories and the composite reference is summarized in Table S2.

Proteomic profiling combined with PCA demonstrated clear spectral separation between the *A. baumannii* isolates and control spectra. Field isolates clustered tightly with the reference strain, indicating high spectral consistency and species-level discrimination. These results confirm the robustness of MALDI-TOF MS for identifying *A. baumannii* in RTE food matrices (Figure S1).

### 3.4. PCR-Based Detection of Species-Confirmation and Resistance Genes

All composite-confirmed *A. baumannii* isolates were screened by PCR to confirm species identity and characterize carbapenem resistance determinants. Species confirmation was supported by universal detection of the intrinsic *bla*_OXA-51-like_ marker, excluding misassigned members of the ACB complex. Screening of acquired resistance genes showed a clear predominance of OXA-type carbapenemases, with *bla*_OXA-23-like_ representing the dominant determinant. Other OXA-type genes were detected infrequently, and metallo-β-lactamase genes were rare or absent. This pattern indicates that carbapenem resistance in the analyzed isolates was primarily mediated by OXA-type β-lactamases, with only sporadic involvement of metallo-β-lactamases. The distribution of species-confirmation and resistance genes among composite-confirmed isolates is summarized in Table 4.

### 3.5. Antimicrobial Susceptibility Profiles

AST revealed broad and clinically relevant resistance among the 19 composite-confirmed *A. baumannii* isolates. Carbapenem resistance was prominent, with 17 out of 19 isolates resistant to imipenem and 16 out of 19 resistant to meropenem, indicating limited activity of these agents.

**Figure 3:**
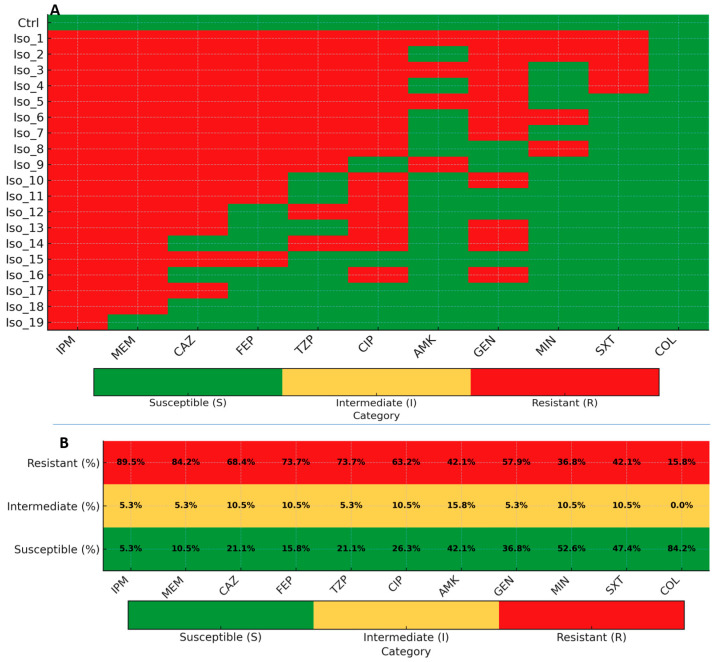
Antimicrobial susceptibility among composite-confirmed *A. baumannii* isolates (*n* = 19). (**A**) Isolate-level heatmap displays susceptibility categories (green = susceptible, yellow = intermediate, red = resistant) for 11 antimicrobial agents. The American Type Culture Collection (ATCC) 19606 strain is included as the fully susceptible control. (**B**) Bar plots show aggregate proportions of resistant, intermediate, and susceptible isolates by agent. Antibiotic abbreviations: IPM (imipenem), MEM (meropenem), CAZ (ceftazidime), FEP (cefepime), TZP (piperacillin–tazobactam), CIP (ciprofloxacin), AMK (amikacin), GEN (gentamicin), MIN (minocycline), SXT (trimethoprim–sulfamethoxazole), COL (colistin). Interpretation based on Clinical and Laboratory Standards Institute (CLSI) M100, 35th edition; colistin was assessed by broth microdilution when performed and not by disk diffusion.

Cephalosporins and β-lactam/β-lactamase inhibitor combinations also showed reduced effectiveness, whereas resistance to ciprofloxacin was common. Aminoglycoside susceptibility varied across isolates, with partial retention of activity for amikacin and gentamicin. Minocycline demonstrated lower resistance overall, with more than half of the isolates remaining susceptible. Colistin retained the highest activity, with 16 out of 19 isolates categorized as susceptible. Detailed susceptibility distributions by agent are summarized in Table 5 and visualized in Figure 3.

Cumulative resistance profiling showed that 17 out of 19 isolates met the criteria for MDR, and 6 isolates were classified as XDR. The multiple antibiotic resistance index (MARI) was calculated for each isolate as the ratio of the number of antibiotics to which the isolate was resistant to the total number of antibiotics tested (MARI = a/b), following established methodology. Higher MARI values corresponded to XDR phenotypes. Individual resistance profiles and MAR classifications are presented in Table 6. Additional method-comparison analyses between disk diffusion and automated testing are provided in the Supplementary Materials (Table S3).

### 3.6. Time-to-Result and Cost

Operational evaluation showed that rapid proteomic and molecular approaches substantially reduced the time to species identification. MALDI-TOF MS and targeted RT-PCR enabled same-day confirmation with minimal hands-on time. Turnaround time for AST varied by platform, with automated testing enabling earlier susceptibility reporting compared with manual disk diffusion. These operational observations support the feasibility of integrated workflows for routine food-sector AMR surveillance.

## 4. Discussion

Finding MDR *A. baumannii* in RTE foods suggests that food may represent a possible non-clinical reservoir for this clinically important pathogen [9]. This organism has been classified as a global threat by the WHO [3,8,58,59]. In the past, *A. baumannii* was mainly linked to hospitals. However, recent evidence shows that it can also survive in the environment. This includes non-clinical niches such as food and water [60,61,62]. The findings in this study show that MDR *A. baumannii* can contaminate RTE foods in regular community settings. This reinforces earlier concerns that *Acinetobacter* bacteria could be an overlooked issue in food safety contexts [9,63].

A step-by-step testing process was used. This process combined classic culturing, automated analysis, rapid identification by MALDI-TOF MS, and genetic tests for confirmation. Nineteen isolates of *A. baumannii* were detected among the 240 tested food products, which corresponded to a prevalence of 7.9%. The organism was detected in samples from all studied cities. It appeared most often in salads and less often in meat and poultry foods. This broad distribution suggests recurrent contamination across multiple RTE food types, which may be related to raw ingredients, handling practices, and environmental persistence rather than isolated contamination events. These prevalence estimates are similar to those reported in other countries for *Acinetobacter* in food. Reports describe *A. baumannii* in vegetables and fruits in Jordan and Portugal [12,13]. It has also been detected in commercial salads carrying AMR traits [64]. Additional reports describe its presence in raw meat, fish, and seafood [11,65,66]. Sandwiches and other snack foods have also been identified as sources of antibiotic-resistant bacteria [67,68,69]. Overall, these findings indicate that contamination of RTE foods with *A. baumannii* is a recurring observation rather than a rare event.

The observed pattern, with salads most frequently affected, is biologically and practically plausible. Salads and vegetable mixes are typically not cooked. They often contain raw ingredients and undergo repeated handling. These factors increase the opportunity for contamination [35,36,37]. RTE meat and poultry products can also become contaminated. This may occur if hygiene or temperature control during preparation and storage is inadequate [33,70]. The presence of *A. baumannii* across both food categories highlights the ecological flexibility of this pathogen. This is consistent with previous reports describing the contamination of vegetables, fish, and meats exposed to contaminated water sources [11,65,66,68,71].

Fast and reliable testing is essential for food safety and AMR surveillance. MALDI-TOF MS enables rapid species identification, often within minutes. It is also relatively low cost [42,72,73]. Previous studies show that MALDI-TOF MS can greatly reduce identification time compared with older methods. At the same time, it maintains high accuracy [74,75,76]. Other automated systems, such as VITEK-2, are also used to identify bacteria and assess AMR profiles. However, these systems may be slower or less precise for *Acinetobacter* species [32,39,41,77,78]. In this study, MALDI-TOF MS provided rapid species-level identification. Molecular testing was used to confirm uncertain or borderline identifications.

Chromogenic media such as CHROMagar *Acinetobacter* are used for the initial screening of *Acinetobacter* spp. in complex food matrices. These media are designed for selective isolation and do not allow for reliable species identification within the ACB complex [38,79]. The VITEK-2 Compact system was therefore used as an intermediate automated phenotypic step, reflecting its routine use in clinical microbiology laboratories [80]. Previous studies have shown that automated phenotypic systems have limited accuracy for species-level identification of ACB complex isolates when compared with MALDI-TOF MS [81]. For this reason, VITEK-2 results were not used for definitive species assignment. Final confirmation of *A. baumannii* was based on MALDI-TOF MS and targeted PCR detection of bla_OXA-51-like_, which together formed the composite reference standard used in this study [17,55,82].

Distinguishing closely related *Acinetobacter* species can be challenging. Certain genetic markers, such as *bla*_OXA-51-like_, are useful for identifying *A. baumannii* [55]. However, reliance on a single marker may not always provide absolute certainty [46,48,49,83,84]. Advanced identification methods, including MALDI-TOF MS, generally perform well. Nevertheless, occasional misclassification can occur. This is more likely when reference databases are incomplete or species are highly similar [85,86,87,88,89]. In the present study, most isolates were clearly identified. Molecular testing helped resolve uncertain cases. This combined approach supports the use of integrated workflows in food microbiology laboratories, in line with expert guidance [29,34,89,90]. In this study, PCR was applied selectively to resolve borderline or ambiguous identifications and was not required when high-confidence species-level identification by MALDI-TOF MS was obtained.

The AMR patterns observed are notable. Most isolates were resistant to carbapenems. All carried the intrinsic *bla*_OXA-51-like_ gene. Many also harbored *bla*_OXA-23-like_. These patterns are consistent with findings from hospitals and environmental studies worldwide [25,26,27,28,50,52,53,91]. Detection of these resistance determinants in food samples indicates that clinically relevant resistance mechanisms are not restricted to healthcare settings. One isolate also carried *bla*_NDM_. This gene has previously been reported in food-related contexts [27,51,92]. Other studies have described food and vegetable samples containing multidrug-resistant strains with multiple resistance genes. These observations support the possibility that such lineages can enter the food system [93]. These findings suggest that food may contribute to the wider circulation of resistance genes across human, animal, and environmental sectors [54,69,71,94].

Traits that have been widely described in the literature for *A. baumannii*, including biofilm formation, tolerance to desiccation, and prolonged survival on abiotic surfaces, may contribute to its persistence in food-related environments. These characteristics were not directly assessed in the present study, but they provide a plausible biological context for the detection of this organism in RTE foods [5,95,96].

Similarly, previous studies have reported the tolerance of *A. baumannii* isolates to commonly used sanitizers [14]; however, sanitizer tolerance was not evaluated here. Environmental investigations have also shown that this organism can persist in water sources. This may allow transfer to food through irrigation or processing activities [16].

Although this study did not examine exposure or health outcomes, the detection of *A. baumannii* in RTE foods is still relevant for vulnerable groups. These include hospitalized patients, immunocompromised individuals, and others who are more susceptible to infection. The concern is greater in situations where RTE foods are consumed without additional heating. For this reason, our findings point to the need for surveillance rather than indicating a measured health risk.

This study has several strengths, including multi-city sampling, coverage of diverse food categories, and the use of standardized laboratory methods [35,37] with AST interpreted according to current guidelines [29,34]. Limitations include the modest number of confirmed isolates, the cross-sectional design, and the absence of direct comparison with clinical isolates. Differences in study design also limit a direct comparison of prevalence estimates across regions [16,78,97]. Despite these limitations, the findings align with growing international and regional evidence that *Acinetobacter* species are increasingly detected in food and environmental sources [9,60,68,69].

The detection of carbapenem-resistant *A. baumannii* in RTE foods supports the inclusion of food sources in national AMR surveillance programs. International organizations, including the WHO, FAO, and WOAH, advocate integrated monitoring across human, animal, and food sectors [6,7,33,58]. Recent reviews highlight the movement of resistant bacteria between these compartments [16,69,71]. Extending surveillance in Saudi Arabia beyond hospital settings to include RTE foods and food-processing environments may strengthen early detection and support risk assessment efforts.

In summary, this study provides a comprehensive assessment of *A. baumannii* in RTE foods in Saudi Arabia. Multiple approaches were used, including culture-based, automated, proteomic, molecular, and antimicrobial susceptibility methods. The detection of carbapenem-resistant and MDR isolates, together with persistence traits described in the literature, supports the consideration of food within the broader ecology of *A. baumannii*. Future studies should include wider geographic coverage, seasonal sampling, and genomic analysis. These efforts will help clarify transmission pathways and guide targeted control strategies.

## 5. Limitations, Strengths, and Future Directions

This study has some important limitations. First, the RTE samples were collected from four cities in the Al-Qassim region during one season (spring). The results may therefore not reflect contamination patterns in other seasons or in other parts of Saudi Arabia. Differences in ingredient sources, storage conditions, food handling, and regional supply chains could all change the risk of contamination. Future work should include multiple seasons and more regions to build a broader epidemiological picture. Second, the number of composite-confirmed isolates was relatively small (n = 19). This limits the precision of subgroup analyses and reduces the ability to detect rare resistance determinants. Similar issues are often seen in environmental and foodborne surveillance studies that focus on rare or emerging resistance genes in low prevalence settings. In addition, this study focused exclusively on *A. baumannii*, and other *Acinetobacter* species detected during initial screening were not further characterized.

Third, the composite reference standard relied on species-level identification by MALDI-TOF MS and/or *bla*_OXA-51-like_ RT-PCR. Although this strategy is specific and widely accepted, some rare or atypical members of the ACB complex may still be misclassified, as reported in recent evaluations. Fourth, the AST workflow depended mainly on Kirby Bauer disk diffusion and the VITEK-2 Compact system. Colistin susceptibility was confirmed by broth microdilution only when available, and newer agents such as cefiderocol were not tested.

Furthermore, phenotypic carbapenemase assays (e.g., modified Hodge test or Carba NP) were not performed. Therefore, carbapenem resistance due to enzymatic mechanisms could not be fully distinguished from non-enzymatic mechanisms such as porin alterations or efflux pump activity. In addition, no data were generated on clonal relatedness or genetic similarity between foodborne isolates and clinical strains. This limits inference regarding transmission pathways and constrains conclusions about direct links to public health impact. Finally, whole-genome sequencing (WGS) was not performed. WGS would have helped to define clonal relatedness, track mobile genetic elements, and describe co-resistance patterns, and it is increasingly viewed as a reference approach for high-resolution epidemiology and resistome analysis in One Health surveillance.

At the same time, the study has clear strengths. The combined use of culture, rapid proteomic identification, and targeted molecular testing provided dependable species-level identification, judged against a pre-defined composite standard. AST data from Kirby Bauer and VITEK-2 were interpreted according to CLSI guidance and supplemented with practical measures such as turnaround time and micro-costs. The multi-city sampling strategy and stratification by food category generated information that is directly useful for risk-based food safety monitoring. The detection of CRAB carrying OXA-23-type enzymes in RTE foods points to a significant One Health concern and suggests that community exposure may occur outside hospitals.

These findings also point to priorities for future work. Integrating WGS will be important for linking foodborne *A. baumannii* isolates with clinical and environmental strains. It will also be important for defining the genetic context of resistance genes such as *bla*_OXA-23_ and *bla*_OXA-24/40_, which are often found on mobile elements including transposon Tn2006 and plasmids [66,92]. Surveillance should be expanded to include a wider range of RTE foods. This is especially important for high-handling items such as salads and delicatessen products. Surveillance should also cover more retail outlets, additional regions, and multiple seasons. Alongside food sampling, environmental sampling can help pinpoint contamination sources. This includes swabbing food-contact surfaces and testing water sources. These activities can guide targeted interventions in processing and retail environments [60,61].

Food microbiology laboratories would also benefit from rapid, integrated diagnostic workflows. Platforms that combine MALDI-TOF MS for fast species identification with multiplex RT-PCR for resistance gene detection could enable near real-time genomic surveillance. When supported by portable sequencing technologies, these platforms could also facilitate outbreak investigation when unusual resistance patterns or clusters appear [60,92]. Targeted control measures should be tested within Hazard Analysis and Critical Control Point (HACCP) frameworks. These measures should focus on sanitizer performance, produce washing steps, and hygiene practices throughout processing and handling. The aim is to reduce contamination and the risk of MDR pathogen transmission via RTE foods [70].

Finally, these advances in genomics and surveillance need to be embedded in a One Health approach. Close collaboration among food safety regulators, public health authorities, clinical services, and agricultural sectors is essential. This collaboration supports timely data sharing, clear risk prioritization, and rapid translation of evidence into policies and practices. Such coordinated efforts are central to limiting the spread of MDR *A. baumannii* in the food supply. They are also critical at the interface between humans, animals, and the environment [58].

## 6. Conclusions

In this study, we found that RTE foods can carry MDR *A. baumannii*, including strains with resistance patterns that are usually associated with serious hospital infections. By using a combination of routine culture, automated systems, MALDI-TOF MS, molecular confirmation, and AST, we showed that this pathogen is detectable in non-clinical food environments. These include RTE, and not only healthcare settings. The detection of OXA-type carbapenemase genes in foodborne isolates indicates that important resistance mechanisms can persist outside hospitals. These mechanisms can also be detected in non-clinical environments such as the food chain. These findings highlight surveillance relevance rather than risk quantification. This is because consumer exposure and transmission pathways were not assessed. RTE foods and food-handling environments remain underrepresented in routine AMR surveillance, and their inclusion may strengthen One Health monitoring across food, environmental, and clinical sectors. Future studies that involve larger sample sizes, cover different seasons and regions, and use WGS will help clarify links between food, environmental, and clinical reservoirs. Overall, this work supports a One Health surveillance perspective and emphasizes the value of integrating food-sector data into national AMR monitoring strategies.

## Figures and Tables

**Table 1:** Primer panels used for molecular species confirmation and carbapenem resistance gene detection by SYBR Green real-time PCR on the 7500 Fast platform.

Target Gene	Primer Name	Sequence (5′→3′)	Amplicon (bp)	Annealing Temperature (°C)	Ref.
Primers used for molecular species confirmation					
*bla*_OXA-51-like_	OXA51-F	TAATGCTTTGATCGGCCTTG	353	60	[48]
	OXA51-R	TGGATTGCACTTCATCTTGG			
*gyrB*	Sp4F	CACGCCGTAAGAGTGCATTA	294	60	[49]
	Sp4R	AACGGAGCTTGTCAGGGTTA			
*rpoB* (Zone-1)	Ac696F	TAYCGYAAAGAYTTGAAAGAAG	~350	55–60	[47]
	Ac1093R	CMACACCYTTGTTMCCRTGA			
*rpoB* (Zone-2)	Ac1055F	GTGATAARATGGCBGGTCGT	~450	55–60	[47]
	Ac1598R	CGBGCRTGCATYTTGTCRT			
Primers used for detection of carbapenem resistance genes					
*bla*_OXA-23-like_	OXA23-F	GATCGGATTGGAGAACCAGA	501	56–57	[48]
	OXA23-R	ATTTCTGACCGCATTTCCAT			
*bla*_OXA-24/40-like_	OXA24-F	GGTTAGTTGGCCCCCTTAAA	668	53	[50]
	OXA24-R	AGTTGAGCGAAAAGGGGATT			
*bla*_OXA-58-like_	OXA58-F	AAGTATTGGGGCTTGTGCTG	404	53	[50]
	OXA58-R	CCCCTCTGCGCTCTACATAC			
*bla*_NDM_	NDM-F	GGTTTGGCGATCTGGTTTTC	621	54–56	[51]
	NDM-R	CGGAATGGCTCATCACGATC			
*bla*_VIM_	VIM-F	GATGGTGTTTGGTCGCATA	390	49–52	[52,53]
	VIM-R	CGAATGCGCAGCACCAG			
*bla*_IMP_	IMP-F	GGAATAGAGTGGCTTAAYTCTC	232	52	[54]
	IMP-R	GGTTTAAYAAAACAACCACC			

**Table 2:** Summary of study workflow, from sampling through to final AST and data integration.

Stage	No. of Samples/Isolates	%
Total RTE samples collected	240	100
Growth on selective media	49	20.4
Presumptive *Acinetobacter* spp.	43	17.9
VITEK-2 ACB complex calls	27	11.3
MALDI log(score) ≥ 2.0	21	8.8
RT-PCR (+for *bla*_OXA-51-like_)	19	7.9
Composite-confirmed *A. baumannii*	19	7.9

**Table 3:** Prevalence of composite-confirmed *A. baumannii* among RTE samples (n = 240).

Stratum	Level	No. Tested	Positive (*n*)	Positive (%)	CI (95%)
A. Overall prevalence	Overall	240	19	7.9%	4.9–12.1%
B. By city	Buraydah	60	6	10.0%	3.8–20.5%
	Unaizah	60	5	8.3%	2.8–18.4%
	Al-Bukayriyah	60	4	6.7%	1.8–16.2%
	Ar Rass	60	4	6.7%	1.8–16.2%
C. By food category	Salads	40	6	15.0%	5.7–29.8%
	Meat-based	40	4	10.0%	2.8–23.7%
	Poultry	40	3	7.5%	1.6–20.4%
	Dairy-based	40	2	5.0%	0.6–16.9%
	Rice dishes	40	2	5.0%	0.6–16.9%
	Desserts	40	2	5.0%	0.6–16.9%
	Overall total	240	19	7.9%	4.9–12.1%

**Table 4:** PCR detection of species-confirmation and resistance genes among the composite-confirmed *A. baumannii* isolates (*n* = 19).

Gene	Class	Positive	
		*n*	%
*bla*_OXA-51-like_	Intrinsic	19	100%
*bla*_OXA-23-like_	OXA-CHDL	16	~84%
*bla*_OXA-24/40-like_	OXA-CHDL	2	~10%
*bla*_OXA-58-like_	OXA-CHDL	1	~5%
*bla*_NDM_	MBL	1	~5%
*bla*_VIM_	MBL	0	0%
*bla*_IMP_	MBL	0	0%

**Table 5:** Antimicrobial susceptibility profiles of *A. baumannii* isolates (*n* = 19).

Antibiotic	Class	Resistant		Intermediate		Susceptible	
		*n*	%	*n*	%	*n*	%
Imipenem	Carbapenem	17	89.5%	1	5.3%	1	5.3%
Meropenem		16	84.2%	1	5.3%	2	10.5%
Ceftazidime	Cephalosporin (3rd)	13	68.4%	2	10.5%	4	21.1%
Cefepime	Cephalosporin (4th)	14	73.7%	2	10.5%	3	15.8%
Piperacillin–tazobactam	β-lactam/β-lactamase inhibitor	14	73.7%	1	5.3%	4	21.1%
Ciprofloxacin	Fluoroquinolone	12	63.2%	2	10.5%	5	26.3%
Amikacin	Aminoglycoside	8	42.1%	3	15.8%	8	42.1%
Gentamicin		11	57.9%	1	5.3%	7	36.8%
Minocycline	Tetracycline	7	36.8%	2	10.5%	10	52.6%
Trimethoprim–sulfamethoxazole	Folate inhibitor	8	42.1%	2	10.5%	9	47.4%
Colistin	Polymyxin	3	15.8%	-	-	16	84.2%

Notes: Percentages are based on *n* = 19 isolates. Colistin categorization reflects broth microdilution when performed; disk diffusion was not used for colistin. Intermediate indicates results within the CLSI intermediate range.

**Table 6:** MAR Profiles with MDR/XDR Classification.

Isolate	Resistance Profile	No. Resistant (R)	MAR Index	Classification
1	IPM, MEM, CAZ, FEP, TZP, CIP, AMK, GEN, MIN, SXT	10	0.91	XDR
2	IPM, MEM, CAZ, FEP, TZP, CIP, GEN, MIN, SXT	9	0.82	XDR
3	IPM, MEM, CAZ, FEP, TZP, CIP, AMK, GEN, SXT	9	0.82	XDR
4	IPM, MEM, CAZ, FEP, TZP, CIP, GEN, SXT	8	0.73	XDR
5	IPM, MEM, CAZ, FEP, TZP, CIP, AMK, GEN	8	0.73	XDR
6	IPM, MEM, CAZ, FEP, TZP, CIP, GEN, MIN	8	0.73	XDR
7	IPM, MEM, CAZ, FEP, TZP, CIP, GEN	7	0.64	MDR
8	IPM, MEM, CAZ, FEP, TZP, CIP, MIN	7	0.64	MDR
9	IPM, MEM, CAZ, FEP, TZP, AMK	6	0.55	MDR
10	IPM, MEM, CAZ, FEP, CIP, GEN	6	0.55	MDR
11	IPM, MEM, CAZ, FEP, CIP	5	0.45	MDR
12	IPM, MEM, CAZ, TZP, CIP	5	0.45	MDR
13	IPM, MEM, CAZ, CIP, GEN	5	0.45	MDR
14	IPM, MEM, TZP, CIP, GEN	5	0.45	MDR
15	IPM, MEM, CAZ, FEP	4	0.36	MDR
16	IPM, MEM, CIP, GEN	4	0.36	MDR
17	IPM, MEM, CAZ	3	0.27	MDR
18	IPM, MEM	2	0.18	Non-MDR
19	IPM	1	0.09	Non-MDR

Notes: MDR = non-susceptible to at least one agent in three or more antimicrobial classes; XDR = non-susceptible to at least one agent in all but one or two classes. MAR index = number of antibiotics to which the isolate is resistant (R) divided by the total number tested. Class and agent abbreviations: IPM, imipenem; MEM, meropenem; CAZ, ceftazidime; FEP, cefepime; TZP, piperacillin–tazobactam; CIP, ciprofloxacin; AMK, amikacin; GEN, gentamicin; MIN, minocycline; SXT, trimethoprim–sulfamethoxazole.

## Data Availability

The raw data supporting the conclusions of this article will be made available by the authors on request.

## Supplementary Materials

The following supporting information can be downloaded at: https://www.mdpi.com/article/10.3390/pathogens15030261/s1, Figure S1: MALDI-TOF MS protein spectral profiling and multivariate analysis of reference strains and field isolates; Table S1: Diagnostic performance of identification platforms relative to composite reference; Table S2: MALDI-TOF MS log-score interpretation and agreement with composite reference; Table S3: Pairwise Agreement Between KB and VITEK-2 by Antibiotic Class; Table S4: Presumptive *Acinetobacter* isolates excluded during the stepwise confirmation workflow.
