# Supplementary material for: Antimicrobial Resistance in Acinetobacter baumannii Isolated from Ready-to-Eat Foods in Saudi Arabia
Source: Pathogens. 2026 Mar 1;15(3):261. doi: 10.3390/pathogens15030261 (PMC13029645; doi:10.3390/pathogens15030261)
Supplement: Supplementary file 1 [file pathogens-15-00261-s001.zip › pathogens-4115619-supplementary.pdf]

Table S1. Diagnostic performance of identification platforms relative to composite reference.

Identification method	TP	FN	FP	TN	Sensitivity (%)	Specificity (%)	PPV (%)	NPV (%)
MALDI-TOF MS	19	0	2	27	100.0 (95% CI, 82.4-100)	93.1 (95% CI, 77.2-99.2)	90.5 (95% CI, 69.6-98.8)	100.0 (95% CI, 87.2-100.0)
VITEK-2 Compact	16	3	4	25	84.2 (95% CI, 60.4-96.6)	86.2 (95% CI, 68.3-96.1)	80.0 (95% CI, 56.3-94.3)	89.3 (95% CI, 71.8-97.7)
RT-PCR (*bla*$_{OXA\text{-}51\text{-}like}$)	19	0	0	29	100.0 (95% CI, 82.4-100)	100.0 (95% CI, 88.1-100)	100.0	100.0

Abbreviations: TP: True Positive; FP: False Positive; TN: True Negative; FN: False Negative; PPV (%): Positive Predictive Value; NPV (%): Negative Predictive Value. Note: Composite reference = MALDI-TOF MS score ≥ 2.0 and/or RT-PCR positive for *blaOXA-51-like*.

Table S2. MALDI-TOF MS log-score interpretation and agreement with composite reference.

MALDI-TOF MS category	Interpretation	No. of isolates (%)	Agreement with composite reference
≥ 2.0	Species-level confidence	21 (77.8%)	19/21 (90.5%)
1.70–1.99	Genus-level confidence	6 (22.2%)	2/6 (33.3%)
< 1.70	Unreliable identification	0 (0%)	-
Overall	-	-	98% overall accuracy

Note: Composite reference = MALDI-TOF MS score ≥ 2.0 and/or RT-PCR positive for *bla*OXA-51-like.

Table S3. Pairwise Agreement Between KB and VITEK-2 by Antibiotic Class.

Antibiotic Class (Examples)	CA	Predominant Discrepancy Pattern	Notes
Carbapenems (IPM, MEM)	Yes	Rare mE near breakpoints	High resistance; robust concordance
Cephalosporins (CAZ, FEP)	Yes	mE at I boundaries; occasional ME	Borderline zone/MIC behavior
β-lactam/β-lactamase inhibitors (TZP)	Yes	Occasional mE	Similar to cephalosporins
Fluoroquinolones (CIP)	Yes	mE at S/I or R/I edge	Heterogeneous mechanisms
Aminoglycosides (AMK, GEN)	Yes	mE with mixed S/I patterns	Combination therapy advisable
Tetracycline (MIN)	Yes	Infrequent mE	Best non-polymyxin activity
Folate inhibitor (SXT)	Yes	mE from edge-reading	KB 80% edge rule
Polymyxin (COL)	N/A	-	MIC only; diffusion not valid

Table S4. Presumptive *Acinetobacter* isolates excluded during the stepwise confirmation workflow

Workflow stage	No. of isolates	Identification outcome	Reason for exclusion from final analysis
Presumptive *Acinetobacter* spp. after selective culture and basic phenotypic screening	43	Presumptive *Acinetobacter* spp.	Initial screening pool prior to confirmatory identification
Not assigned to ACB complex by VITEK-2 Compact	16	Non-ACB complex identification	Did not meet phenotypic criteria for ACB complex
Assigned to ACB complex by VITEK-2 Compact	27	ACB complex (phenotypic identification)	Proceeded to confirmatory testing
Failed composite confirmation (MALDI-TOF MS score < 2.0 and RT-PCR $bla_{\text{OXA-51-like}}$ negative)	8	Non-confirmatory results	Did not meet composite reference standard
Composite-confirmed *A. baumannii*	19	Confirmed by MALDI-TOF MS ≥ 2.0 and/or RT-PCR	Included in prevalence and AMR analyses

Footnote: Presumptive isolates were not assigned species-level identities unless confirmed by the predefined composite reference standard. Isolates failing confirmatory criteria were classified as non-*A. baumannii* and excluded from downstream analyses to avoid overclassification.

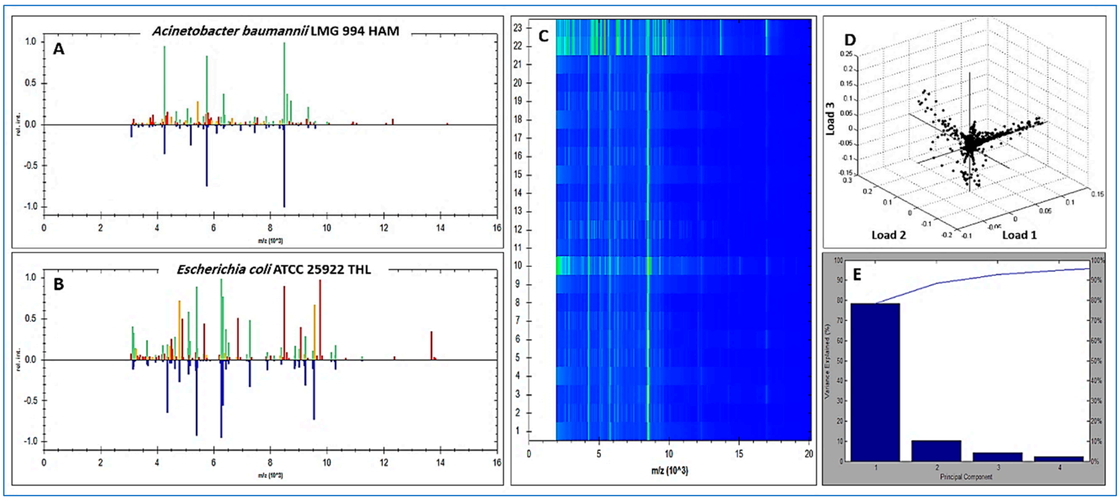

Figure S1. MALDI-TOF MS protein spectral profiling and multivariate analysis of reference strains and field isolates. (A) Overlay of the protein spectra from *Acinetobacter baumannii* LMG 994 HAM reference strain and a matched field isolate, illustrating strong spectral congruence. (B) Protein spectra of *Escherichia coli* ATCC 25922 THL aligned with the Bacterial Test Standard (BTS) used as positive control and calibrator. In panels A and B, blue peaks at the lower section correspond to the deposited reference spectra, while green peaks above indicate highly confident matches; yellow and red peaks represent intermediate and low-confidence or mismatched signals, respectively. (C) Gel view displaying aligned mass-to-charge (m/z) features across all spectra, highlighting the distribution and intensity of discriminatory protein peaks (lanes 1 to 21 correspond to *A. baumannii* isolates, and lanes 22 and 23 are duplicate BTS calibration samples). (D) Three-dimensional principal component analysis (3D-PCA) score plot depicting distinct clustering of spectra based on species-specific protein fingerprints, confirming species-level differentiation. (E) Scree plot illustrating the percentage of variance explained by the first four principal components, with the first component (PC1) accounting for the majority of spectral variance.
